# NADPH Oxidase 4 Contributes to Myoblast Fusion and Skeletal Muscle Regeneration

**DOI:** 10.1155/2019/3585390

**Published:** 2019-11-18

**Authors:** Tae Hyun Youm, Sun-Hee Woo, Eun-Soo Kwon, Sung Sup Park

**Affiliations:** ^1^Aging Research Center, Korea Research Institute of Bioscience and Biotechnology, 125 Gwahak-ro, Yuseong-gu, Daejeon 34141, Republic of Korea; ^2^Laboratory of Physiology, College of Pharmacy, Chungnam National University, 99 Daehak-ro Yuseong-gu, Daejeon 34134, Republic of Korea

## Abstract

Myoblast fusion is an essential step in skeletal muscle development and regeneration. NADPH oxidase 4 (Nox4) regulates cellular processes such as proliferation, differentiation, and survival by producing reactive oxygen species (ROS). Insulin-like growth factor 1 induces muscle hypertrophy via Nox4, but its function in myoblast fusion remains elusive. Here, we report a ROS-dependent role of Nox4 in myoblast differentiation. Regenerating muscle fibers after injury by cardiotoxin had a lower cross-sectional area in *Nox4*-knockout (KO) mice than myofibers in wild-type (WT) mice. Diameters and fusion index values of myotubes differentiated from *Nox4*-KO primary myoblasts were significantly lower than those of myotubes derived from WT myoblasts. However, no difference was observed in the differentiation index and expression of MyoD, myogenin, and myosin heavy chain 3 (MHC) between KO and WT myotubes. The decreased fusion index was also observed during differentiation of primary myoblasts and C2C12 cells with suppressed Nox4 expression. In contrast, in C2C12 cells overexpressing Nox4, the fusion index was increased, whereas the differentiation index and MHC and myogenin protein expression were not affected compared to control. Interestingly, the expression of myomaker (Tmem8c), a fusogenic protein that controls myoblast fusion, was reduced in Nox4-knockdown C2C12 cells. The myomaker expression level was proportional to the cellular ROS level, which was regulated by of Nox4 expression level. These results suggests that Nox4 contributes to myoblast fusion, possibly through the regulation of myomaker expression via ROS production, and that Nox4-dependent ROS may promote skeletal muscle regeneration and growth.

## 1. Introduction

NADPH oxidase 4 (Nox4) is expressed in various fiber types of skeletal muscle, such as gastrocnemius and soleus muscles [1]. Nox4 increases oxidative stress on skeletal muscle ryanodine receptor (RyR) in metastases, thus inducing skeletal muscle weakness in breast cancer [2]. In normal physiology, reactive oxygen species (ROS) produced by Nox4 in the sarcoplasmic reticulum induce oxidation of cysteine thiols of RyR1, leading to an increase in its activity in cultured myofibers and enhanced contractile performance in intact muscle [3]. However, enhanced binding of Nox4 to RyR1 induces excessive oxidation and nitrosylation, and reduces muscle force in skeletal muscle after spinal cord injury [4]. Nox4 expression is increased during differentiation [5] as well as by physical exercise [1]. However, the role of Nox4 in muscle force remains unclear. Nox4-mediated ROS does not contribute to the decline in skeletal muscle force during or after induction of fatigue in mice [6]. Fiber-type switch from fast-twitch to intermediate fibers is induced by voluntary running exercise even in Nox4-deficient mice [7]. Nox4-derived ROS contribute to an increase in muscle mass by promoting receptor phosphorylation by insulin-like growth factor 1 (IGF-1), leading to myocyte hypertrophy in C2C12 cells [5].

Myoblast fusion is a multistep process that involves numerous proteins and is required for muscle development during embryogenesis and muscle regeneration in adults [8, 9]. An essential step in this process is cytoskeletal rearrangement, which involves many components, such as N-Wasp, Rac1, Cdc42, and Dock180 [10–12]. A recent study showed that myomaker (Tmem8c) regulates myoblast fusion without impacts on differentiation potential mediated by myosin protein expression in mice [13]. Unlike other Tmem8 proteins (Tmem8a and Tmem8b), myomaker (*Mymk*) has fusogenic activity [14, 15]. Myomaker function is conserved in zebrafish [16], chickens [17], and humans [18]. Minion (myomerger), another fusogenic micropeptide, regulates cytoskeletal rearrangement in myoblast membrane fusion independent of myomaker. Myomaker mediates the merging of the outer leaflets of membranes, whereas minion is required for the merging of the inner leaflets to form the fusion pore [19]. Myomaker, not minion, endows fibroblasts with the capability of cell-to-cell fusion with myoblasts [13, 20], and heterologous expression can promote fibroblast fusions [20–22].

A few studies have investigated the regulatory mechanisms of myomaker expression. Muscle-specific transcription factors, MyoD and myogenin, promote chicken myoblast fusion through binding to the E-Box region of the myomaker promoter [17]. microRNAs, miR-140-3p and miR-491, negatively regulate myomaker expression, resulting in the inhibition of myogenic differentiation [17, 23]. In this study, we investigated the role of Nox4 expression and Nox4-mediated ROS in skeletal muscle differentiation. Myoblast fusion was analyzed using primary myoblasts derived from *Nox4* knockout (KO) or wild-type (WT) mice. Fusion efficiency was also determined in C2C12 cells after Nox4 expression was suppressed or promoted. Finally, we evaluated whether myomaker expression depends on Nox4 expression and ROS generation.

## 2. Materials and Methods

### 2.1. Animals

C57BL/6 mice were purchased from the Laboratory Animal Resource Center of Korea Research Institute of Bioscience and Biotechnology (KRIBB). *Nox4*-KO mice were generated as previously described [24]. All animal experiments were carried out in strict accordance with the protocols approved by the Animal Care and Use Committee of KRIBB.

### 2.2. Muscle Regeneration Assay

Cardiotoxin (CTX, Sigma) injury and muscle regeneration assay followed a previously described procedure [25]. Briefly, 50 *μ*L of CTX (20 *μ*M) was injected into the tibialis anterior (TA) muscles of 3-month-old WT and *Nox4*-KO mice. TA muscles harvested at 0, 7, and 14 days post-injection were fixed in 4% paraformaldehyde. Frozen sections (8 *μ*m thick) were stained with hematoxylin and eosin (H&E) and analyzed under a fluorescence microscope. Cross-sectional areas (CSAs) were measured using NIH ImageJ software (http://rsb.info.nih.gov/ij).

### 2.3. Isolation and Culture of Mouse Primary Myoblasts

Primary myoblasts were isolated from TA muscles of 3-month-old mice as previously described [25]. Briefly, TA muscles were dissected and incubated in dissociation buffer containing 2.4 U/mL dispase II (Roche), 1% collagenase D (Roche), and 2.5 *μ*M CaCl_2_ at 37°C for 20 min. The triturated slurry was passed through a 70-*μ*m mesh (BD Biosciences) to eliminate debris. Cells were collected and resuspended in growth medium containing Ham's F-10 (Gibco), 20% fetal bovine serum (FBS, Gibco), antibiotics (Gibco), and 5 ng/mL basic fibroblast growth factor (Promega). To remove fibroblasts, after the cells were plated on noncoated plates and incubated for 1 h, the floating myoblasts were transferred onto collagen-coated culture dishes. Myoblasts from WT or *Nox4*-KO mouse donors were cultured for the same number of passages and used in all experiments. For myoblast differentiation, cultured cells (1 × 10^5^) were transferred into differentiation medium (DM) consisting of high-glucose Dulbecco's modified Eagle's medium (DMEM, Gibco) supplemented with antibiotics and 5% horse serum (Gibco). C2C12 myoblasts were purchased from the American Type Culture Collection and were maintained in growth medium consisting of DMEM supplemented with antibiotics and 10% FBS. To induce differentiation of C2C12 myoblasts, cells (1 × 10^5^) were grown to 90% confluence in growth medium and incubated in DM for 3 days.

### 2.4. Immunoblot Analysis

Muscle tissues and collected myoblasts were homogenized and lysed in RIPA buffer (Thermo Fisher Scientific) containing protease inhibitor cocktails (P2714, Sigma-Aldrich). The lysates were centrifuged and 20–30 *μ*g protein was resolved by sodium dodecyl sulfate-polyacrylamide gel electrophoresis and transferred onto a polyvinylidene fluoride membrane (Millipore). Immunoblot analysis was conducted using antibodies against Nox4 (Ewha Women's University), *α*-tubulin (Abcam), MyoD (Santa Cruz Biotechnology), myosin heavy chain 3 (MHC, Santa Cruz Biotechnology), myogenin (Santa Cruz Biotechnology), myomaker (Abcam, ab188300), and GAPDH (Santa Cruz Biotechnology). HRP-conjugated goat antibody (Santa Cruz Biotechnology) was used as secondary antibody. Immunocomplexes were visualized using Western Blotting Luminol Reagent (Bio-Rad).

### 2.5. Differentiation and Fusion Assays

Differentiation and fusion efficiencies were evaluated as previously described [26], with slight modification. Primary myoblasts and C2C12 cells were differentiated in DM for 2–3 days and myotubes were fixed with PBS containing 4% paraformaldehyde at room temperature for 10 min. Cells were permeabilized with 0.2% Triton X-100 in PBS for 15 min and blocked with 1% bovine serum albumin (in PBS) for 1 h, then incubated with mouse anti-MHC antibody at 4°C overnight and subsequently with goat anti-mouse Alexa Fluor 488 (Thermo Fisher Scientifics) at room temperature for 1 h. Myotubes were also stained with Vectashield containing 4′,6-diamidino-2-phenylindole (DAPI, Vector Laboratories). Samples were analyzed under an Eclipse Ti fluorescence microscope (Nikon). For myotube diameter quantification, the midpoints of the long tube axis of 100–120 MHC-positive myotubes were analyzed using the Nikon NIS Elements software in three independent experiments. For analysis of nuclear numbers, nuclei fractions were calculated as the ratio of nuclei present in MHC-positive myotubes with indicated number of nuclei to total nuclei within MHC-positive myotubes. The differentiation index was determined as the percentage of nuclei in MHC-positive cells among total nuclei. For primary myoblasts, the fusion index was determined as the percentage of nuclei in MHC-positive myotubes (≥3 nuclei) among total nuclei within MHC-positive myotubes. For C2C12 cells, the fusion index was calculated as the percentage of nuclei in MHC-positive myotubes (≥10 nuclei) to total nuclei within MHC-positive myotubes [20]. At least 3 field images were analyzed per experimental group.

### 2.6. Knockdown and Overexpression of Nox4

For knockdown of *Nox4*, siRNAs (50 *μ*M) targeting *Nox4* (nt 27–51) (GGCCAACGAAGGGGUUAAACACCUC, Sigma-Aldrich) were used. AccuTarget Negative Control siRNA (Bioneer, Korea) was used as a control. Myoblasts were seeded in 6-well plates and transfected with siRNAs using RNAi Max Transfection Reagent (Invitrogen) according to the manufacturer's protocol. Six hours after transfection, the medium was replaced with growth medium. For overexpression studies, C2C12 cells were infected with adenovirus expressing mouse Nox4 (Ad-Nox4, Vector Biolabs) at a multiplicity of infection (MOI) of 10 for 24 h and then incubated in DM for 3 days.

### 2.7. Quantitative Reverse-Transcription PCR (RT-qPCR)

Total RNA was extracted from mouse skeletal muscles or cultured cells using TRIzol (Invitrogen) according to the manufacturer's protocol, and 1 *μ*g of RNA was used for first-strand cDNA synthesis (iScript cDNA synthesis kit, Bio-Rad). qPCRs were run in a StepOnePlus RT-PCR System (Applied Biosystems) using a reaction mixture containing cDNA, primers, and SYBR Master Mix according to the manufacturer's protocol. The following primers were used: *Nox4*, 5′-ACTTTTCATTGGGCGTCCTC-3′ and 5′-GAACTGGGTCCACAGCAGAA-3′; *Mymk* 5′-ATCGCTACCAAGAGGCGTT-3′ and 5′-CACAGCACAGACAAACCAGG-3′; *36B4*, 5′-AGATTCGGGATATGCTGTTGG-3′ and 5′-AAAGCCTGGAAGAAGGAGGTC-3′. The 2*Δ*ΔCt method was used to measure relative gene expression after normalization to the mRNA level of *36B4*.

### 2.8. Intracellular ROS Measurement

Intracellular ROS levels were measured using 2,7-dichlorofluorescein diacetate (DCF-DA). C2C12 cells were cultured in 96-well plate for 24 h and treated with siRNAs (50 *μ*M), adenovirus (MOI = 10), the Nox1/4 selective inhibitor GKT137831 (5 *μ*M, Cayman Chemical), or the ROS inhibitor N-acetylcysteine (NAC, 1 mM, Sigma-Aldrich) for 24 h. Then, the cells were incubated in DM for 72 h and washed with Hanks' balanced salt solution (HBSS, Gibco). Myotubes were incubated with 10 *μ*M of DCF-DA solution in the dark at 37°C for 30 min. To remove extracellular ROS, cells were washed with 0.1 mL of HBSS at least three times. Fluorescence was measured using VICTOR X3 (Perkin Elmer) at an excitation wavelength of 488 nm and an emission wavelength of 535 nm.

### 2.9. Statistical Analysis

All experiments were conducted at least three times independently. Quantitative data are presented as the mean ± standard deviation (SD). Means were compared using Student's unpaired *t*-test. Values of *p* < 0.05 were considered statistically significant.

## 3. Results

### 3.1. Nox4 Enhances Skeletal Muscle Regeneration

**Figure 1:**
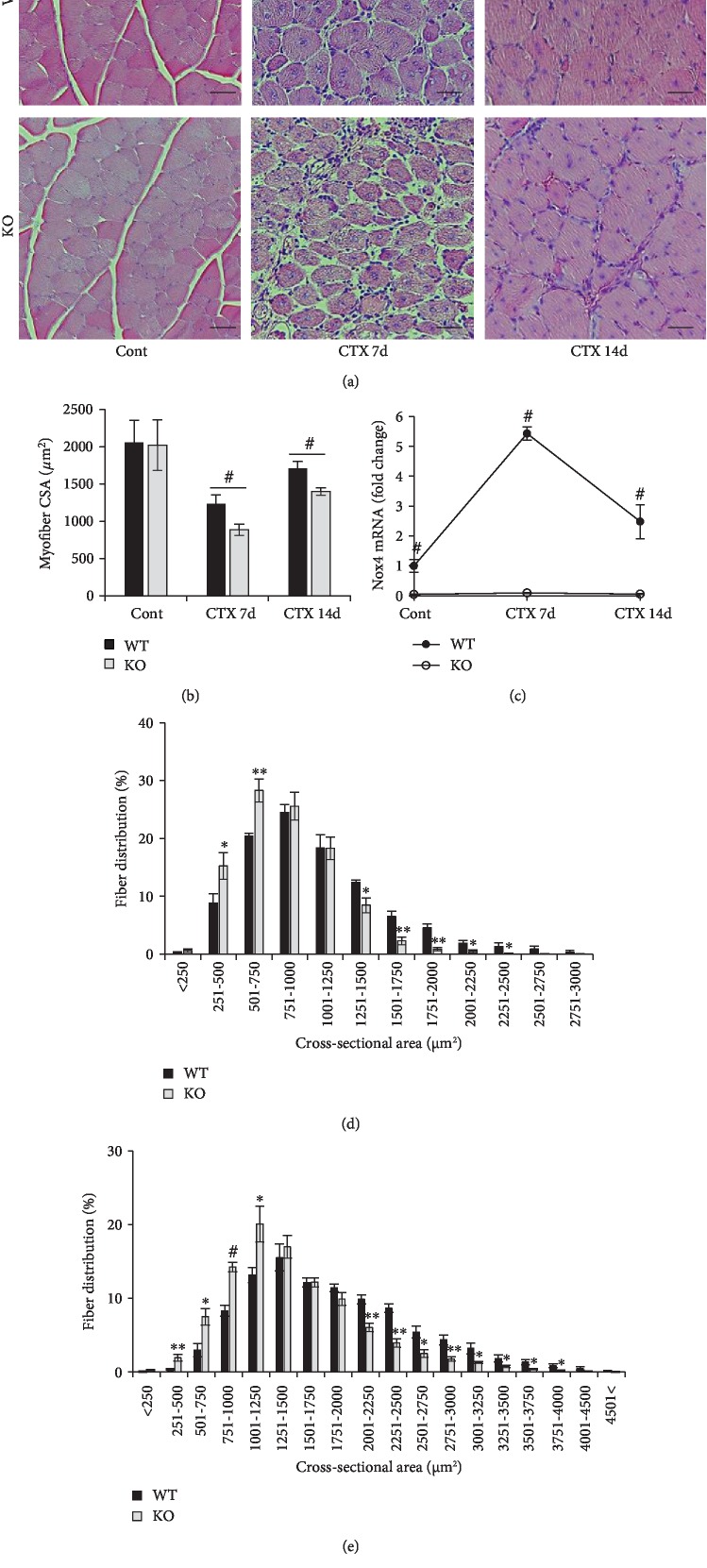
Nox4 contributes to skeletal muscle regeneration. (a) H&E stained sections of TA muscles from WT and *Nox4*-KO mice at 0, 7, and 14 days after 50 *μ*L of CTX (20 *μ*M) was injected. Scale bar, 50 *μ*m. (b) CSAs of regenerating myofibers were analyzed at 0, 7, and 14 days after CTX injection. ^#^*p* < 0.001 (*n* = 5). (c) *Nox4* mRNA levels in TA muscles of WT and KO mice during regeneration after CTX injury was determined by RT-qPCR, using *36B4* for normalization. ^#^*p* < 0.001. The distribution of fiber sizes was analyzed at 7 days (d) and 14 days (e) after CTX injection. ^∗^*p* < 0.05, ^∗∗^*p* < 0.01, ^#^*p* < 0.001 (*n* = 5).

To investigate the role of Nox4 in muscle regeneration, muscle injury was induced by injection of CTX into the TA muscle of 3-month-old WT and *Nox4*-KO mice. CSAs of newly formed KO myofibers at 7 and 14 days were reduced by 28.0% and 18.0% compared to those of newly formed WT myofibers, respectively (Figures 1(a) and 1(b)). No difference was observed in non-injured myofibers between KO and WT animals. In agreement with these results, significant differences in fiber distribution were observed (Figures 1(d) and 1(e)); regenerating muscles in KO mice had smaller myofibers and fewer large myofibers than those in WT mice at 7 and 14 days after injury. *Nox4* mRNA levels in WT muscles were increased by more than 5-fold at 7 days after injury, whereas KO muscles did not express *Nox4* mRNA during regeneration (Figure 1(c)). These results suggested that *Nox4* deficiency impairs muscle regeneration after CTX injury.

### 3.2. Nox4 Is Required for Primary Myoblast Fusion

**Figure 2:**
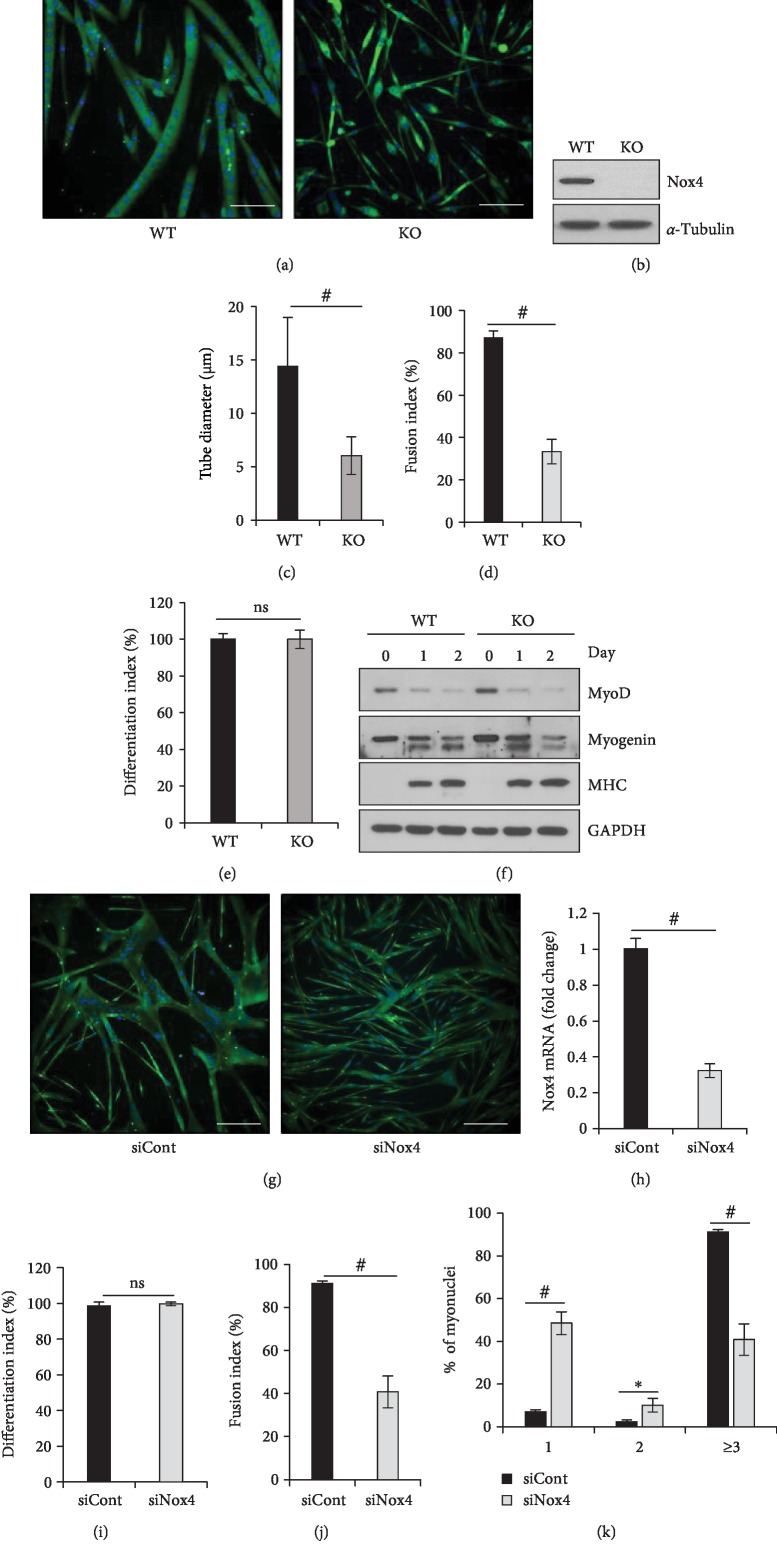
Nox4 is required for myoblast fusion of skeletal muscle. (a) Immunofluorescence images after primary myoblasts derived from WT and *Nox4-*KO mice were cultured in DM for 48 h and stained against MHC (green) and with DAPI for the nucleus (blue). Scale bar, 50 *μ*m. (b) Nox4 expression was analyzed by immunoblotting. (c) Diameters of myotubes were measured using Nikon software. ^#^*p* < 0.001 (*n* = 3). (d) Fusion index values for the experiments done in a were calculated as the percentage of nuclei in MHC-positive myotubes containing ≥3 nuclei among total nuclei in MHC-positive myotubes. ^#^*p* < 0.001 (*n* = 3). (e) Differentiation index values for the experiments in a were quantified as the percentage of nuclei in MHC-positive cells among total nuclei. (f) The expression of MyoD, myogenin, and MHC proteins during differentiation was analyzed by immunoblotting in WT and *Nox4*-KO primary myoblasts. (g) Immunofluorescence images of primary myotubes cultured in DM for 48 h after cells were transfected with siCont or siNox4 and strained for MHC (green) and with DAPI (blue). Scale bar, 50 *μ*m. (h) *Nox4* mRNA levels as evaluated by RT-qPCR, using *36B4* for normalization. ^#^*p* < 0.001 (*n* = 3). (i) Fusion index values for the experiments done in g were calculated as the percentage of nuclei in MHC-positive myotubes containing ≥3 nuclei among total nuclei within MHC-positive myotubes. ^#^*p* < 0.001 (*n* = 3). (j) Differentiation index values for the experiments in g were quantified as the percentage of nuclei in MHC-positive cells among total nuclei. ^#^*p* < 0.001 (*n* = 3). (k) Percentages of nuclei present in MHC-positive myotubes with the indicated number of nuclei were calculated in the experiment done in g. ^∗^*p* < 0.05, ^#^*p* < 0.001 (*n* = 3). ns, no significant difference.

To examine *in-vitro* differentiation, we isolated primary myoblasts from TA muscles of WT and *Nox4*-KO mice. Primary myoblasts were differentiated in DM for 48 h and myotubes were immunostained for MHC. KO myotubes were significantly smaller than WT myotubes (Figures 2(a) and 2(b)). The tube diameter and fusion index of KO myotubes were significantly decreased by 58.3% and 61.7%, respectively, when compared to those of WT myotubes (Figures 2(c) and 2(d)). However, the differentiation index was not affected by *Nox4* KO (Figure 2(e)). Accordingly, the expression of the skeletal muscle differentiation markers MyoD, myogenin, and MHC was not affected by *Nox4* KO (Figure 2(f) and Supplementary Figures [Supplementary-material supplementary-material-1]). To confirm the role of Nox4 in primary myoblast fusion, primary myoblasts from WT mice were treated with Nox4 siRNA (siNox4) and then incubated in DM for 2 days (Figures 2(g) and 2(h), Supplementary Figures [Supplementary-material supplementary-material-1]). The results showed that the fusion index was significantly decreased (by 55.3%) in siNox4 myotubes compared to control siRNA (siCont) myotubes (Figure 2(i)). Again, the differentiation index was not affected (Figure 2(j)). For siNox4 myotubes, the percentage of mononuclei in MHC-positive cells among total nuclei was significantly increased (~7-fold) (Figure 2(k)). These results revealed that ablation of Nox4 suppresses myotube formation through primary myoblast fusion, without affecting the differentiation index.

### 3.3. Nox4 Promotes Myoblast Fusion in C2C12 Cells

**Figure 3:**
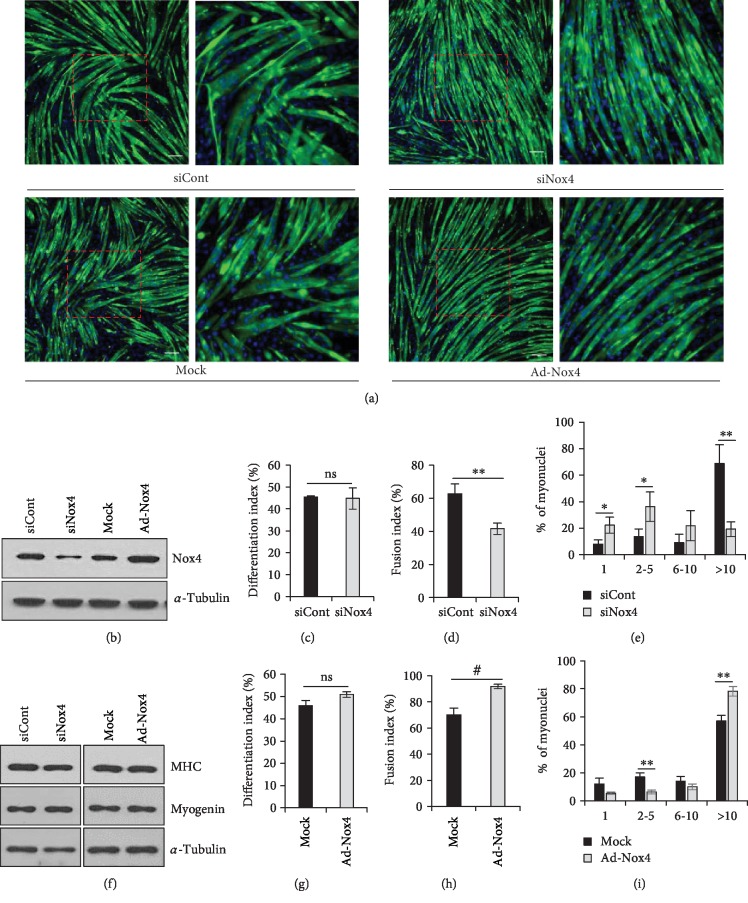
Nox4 enhances myoblast fusion in C2C12 cells. (a) Immunofluorescence images of *Nox4*-KD or -overexpressing C2C12 cells cultured in DM for 3 days after staining for MHC (green) and with DAPI (blue). Scale bar, 50 *μ*m. (b) Protein levels of Nox4 as analyzed by immunoblotting in *Nox4*-KD or -overexpressing C2C12 cells. (c) Differentiation index values were calculated as the percentage of nuclei in MHC-positive cells to total nuclei in *Nox4*-KD cells (siCont and siNox4) (*n* = 3). (d) Fusion index values were calculated as the percentage of nuclei present in MHC-positive myotubes (≥10 nuclei) to total nuclei in MHC-positive myotubes among *Nox4*-KD cells. ^∗∗^*p* < 0.01 (*n* = 3). (e) Percentages of nuclei in MHC-positive myotubes with the indicated number of nuclei were calculated in *Nox4*-KD cells. ^∗^*p* < 0.05, ^∗∗^*p* < 0.01 (*n* = 3). (f) Protein levels of MHC and myogenin as analyzed by immunoblotting in *Nox4*-KD or -overexpressing C2C12 cells. (g) Differentiation index values were calculated as the percentage of nuclei in MHC-positive cells to total nuclei in Nox4-overexpressing cells (mock and Ad-Nox4) (*n* = 3). (h) Fusion index values were calculated as the percentage of nuclei present in MHC-positive myotubes (≥10 nuclei) to total nuclei in MHC-positive myotubes among Nox4-overexpressing cells. ^#^*p* < 0.001 (*n* = 3). (i) Percentages of nuclei in MHC-positive myotubes with the indicated number of nuclei were calculated in Nox4-overexpressing cells. ^∗∗^*p* < 0.01 (*n* = 3). ns, no significant difference.

To evaluate the role of Nox4 in myoblast fusion further, we transfected C2C12 cells with siNox4 or Ad-Nox4 for Nox4 knockdown or overexpression, respectively. *Nox4*-knockdown (*Nox4*-KD) significantly reduced myotube size, without affecting the differentiation index and MHC and myogenin expression (Figures 3(a)–3(c), 3(f), and Supplementary Figures [Supplementary-material supplementary-material-1]). However, the fusion index was decreased by 28.8% when compared to that of siCont-transfected cells (Figure 3(d)). For myotubes derived from *Nox4*-KD cells, the percentage of low number of nuclei (1–5 nuclei) in MHC-positive myotubes was increased, whereas the percentage of multi-nuclei (>10 nuclei) in myotubes was reduced compared to that in siCont myotubes (Figure 3(e)). Conversely, overexpression of Nox4 (Figure 3(b)) increased the fusion index, whereas the differentiation index and MHC and myogenin expression were not affected (Figures 3(a), 3(f)–3(h)). The fusion index was increased by 31.0% in Ad-Nox4-transfected myotubes compared to that in mock-treated cells. The percentage of low number of nuclei (2–5 nuclei) was decreased and that of multi-nuclei (≥10 nuclei) was increased in Nox4-overexpressed large myotubes (Figure 3(i)). Gene rescue in *Nox4*-KO primary myoblasts showed slightly improved myoblast fusion (Supplementary [Supplementary-material supplementary-material-1]). These results suggested that Nox4 expression regulates myoblast fusion during C2C12 myoblast differentiation.

### 3.4. Nox4-Mediated ROS Contributes Myomaker Expression

**Figure 4:**
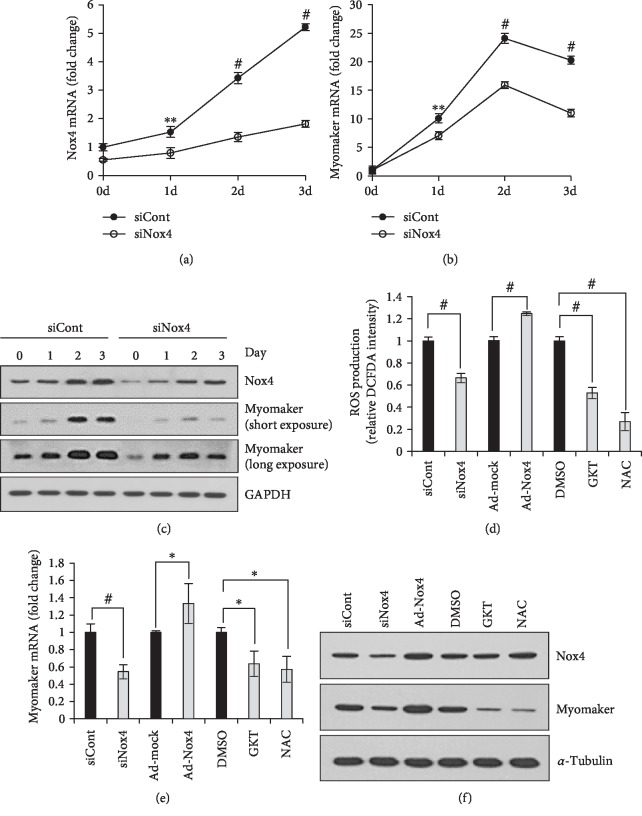
Myomaker expression depends on Nox4-mediated ROS. After C2C12 cells were treated with either siCont or siNox4, mRNA levels of *Nox4* (a) and *Mymk* (b) during differentiation were determined by RT-qPCR, using *36B4* for normalization.^∗∗^*p* < 0.01, ^#^*p* < 0.001 (*n* = 3). (c) Protein levels of Nox4 and myomaker were determined by immunoblotting. Following pretreatment with siNox4, GKT137831 (GKT), or NAC in growth medium, C2C12 cells were incubated in DM for 2 days, ROS production was evaluated by DCF-DA assay (d), the *Mymk* mRNA level was determined by RT-qPCR using *36B4* for normalization (e), and protein levels of Nox4 and myomaker were estimated by immunoblotting (f). Overexpression of Nox4 was achieved by infection with Ad-Nox4 (MOI = 10). ^∗^*p* < 0.05, ^∗∗^*p* < 0.01, ^#^*p* < 0.001 (*n* = 3).

Next, we investigated the expression of several fusion-related proteins, including myomaker, minion, Rac1, and N-Wasp during differentiation of C2C12 cells treated with siNox4 or siCont. The *Nox4* mRNA level was increased by 5.2-fold in siCont-transfected C2C12 cells after 3 days in DM, and this increase was suppressed in siNox4-transfected cells (Figure 4(a)). Concomitantly, *Mymk* expression was decreased in siNox4-transfected compared to siCont-treated control cells (Figure 4(b)). *Mymk* expression was also suppressed in Nox4-KO mouse TA muscle during regeneration (Supplementary [Supplementary-material supplementary-material-1]). However, mRNA levels of *Minion*, *Rac1*, and *N-Wasp* were not affected by *Nox4*-KD (Supplementary Figures [Supplementary-material supplementary-material-1]). Nox4 and myomaker protein levels during differentiation were directly proportional to their respective mRNA levels (Figure 4(c), Supplementary Figures [Supplementary-material supplementary-material-1]). These results suggested that myomaker and Nox4 expression levels are correlated.

We next examined Nox4-dependent intracellular ROS production using a DCF-DA assay. ROS production in Nox4-overexpressing C2C12 cells was increased by 24.5%, whereas ROS production in *Nox4*-KD C2C12 cells was reduced by 33.5% when compared with control cells. GKT137831, Nox1/Nox4 inhibitor, significantly inhibited ROS production when compared to DMSO treatment, and ROS scavenger, NAC, effectively suppressed ROS level (Figure 4(d)). To investigate the role of Nox4-produced ROS in myoblast fusion, we treated C2C12 cells with GKT137831 before differentiation. GKT137831 treatment lowered the myotube size and fusion index compared to vehicle treatment (DMSO), whereas it did not affect the differentiation index (Supplementary Figures [Supplementary-material supplementary-material-1]). Myomaker mRNA expression was dependent on Nox4 expression (was decreased by 42.0% in Nox4-KD cells and increased by 40.1% in Nox4-overexpressing cells) and was also reduced by 36.3% and 42.7% in GKT137831 and NAC treatments, respectively (Figure 4(e)). Protein expression showed similar patterns with mRNA expression (Figure 4(f), Supplementary Figures [Supplementary-material supplementary-material-1]). These results collectively suggested that Nox4-mediated ROS contributes the expression level of the myomaker fusion protein.

## 4. Discussion

The current study revealed that Nox4 contributes to myoblast fusion during differentiation. Myofibers of *Nox4*-KO mice had lower CSAs during regeneration than those of WT mice. However, MyoD, myogenin, and MHC expression were not reduced in *Nox4*-KO myotubes during primary myoblast differentiation. We observed that Nox4 expression increased during differentiation, which is consistent with findings in a previous report [5]. When Nox4 expression was abolished or suppressed in primary myoblasts, differentiation was not affected, but fusion was suppressed. Consistent findings were obtained in C2C12 cells. We could observe myotubes on day 3 in DM, and the expression of myogenic markers were not changed between siCont and siNox4 C2C12 cells, which is consistent with a previous report in the last stage of differentiation [27]. In contrast, overexpression of Nox4 promoted fusion. The concomitant increase of Nox4 expression after differentiation could be the reason why gene rescue in *Nox4*-KO primary myoblasts did not recover fusion efficiency as did WT primary cells because the expression of Nox4 using viral infection did not follow a normal regulation signaling during myoblast differentiation. Taken together, these findings indicate that Nox4 enhances myoblast fusion, without affecting differentiation potential.

The effect of Nox4 expression on the expression of fusion-related proteins was evaluated in C2C12 cells; *Mymk* expression was decreased when *Nox4* expression was suppressed. Myomaker controls myoblast fusion without affecting the differentiation capacity during muscle differentiation in mice and humans [13]. Fibroblasts overexpressing myomaker can fuse with C2C12 myoblasts [13]. However, the regulation of myomaker expression remains unclear. MyoD and myogenin regulate myomaker expression in chicken myoblast fusion [17]. Myomaker expression is regulated by myogenin in mouse and human muscles [28], whereas myoblast fusion is dispensable for skeletal muscle function in zebrafish [29]. Myomaker expression in growth medium was increased with vitamin C treatment, and knockdown of Tet2, a ten-eleven translocation methylcytosine dioxygenase, reduced the expression of myomaker and myogenin without affecting MyoD expression in C2C12 myoblasts [30]. We observed sharply increased myomaker expression during differentiation, consistent with a previous report [13]. However, the expression of myomaker was reduced in differentiated myotubes when Nox4-expression was suppressed and when myoblasts were treated with NAC or GKT137831. A fine-tuned signaling may be involved in the regulation of fusion during myoblast differentiation because gene expression patterns are largely different between before and after differentiation [31].

Nox4-mediated superoxide induces skeletal muscle hypertrophy via a mechanism involving peroxynitrite and a transient receptor potential cation channel [32]. Phenylephrine induces cardiac hypertrophy in mice via oxidation of histone deacetylase mediated by Nox4-dependent ROS [33]. IGF-1-dependent muscle hypertrophy is accompanied by Nox4-dependent ROS generation and IGF-1 receptor phosphorylation [5]. In our study, Nox4-generated ROS promoted fusion. The Nox1/Nox4 inhibitor GKT137831 [34] suppressed fusion, without affecting differentiation. Negligible amount of Nox1 and Nox2 mRNAs was detected in TA muscle and C2C12 cells (data not shown), which is consistent with a previous report [5]. Intracellular ROS levels were proportional to Nox4 expression levels and contributed to myomaker expression levels. NAC treatment significantly reduces fusion in human primary myoblasts [35]. However, it is not known whether forced expression of myomaker increases myoblast fusion in *Nox4*-KO cells, even though myomaker expression level determines fusion performance in fibroblasts [20] and myoblast differentiation [13].

Autophagy activation by ROS can be a possible mediator of myoblast fusion during differentiation. ROS enhances autophagy maturation in mouse coronary arterial myocytes [36]. 7-Ketocholesterol stimulation induces autophagy via ROS generation by Nox4 in vascular and aortic smooth muscle cells [37]. Nox4-dependent ROS promote autophagy during glucose deprivation [38]. Platelet-derived growth factor BB induces autophagy activation via Nox4-mediated ROS [39]. Furthermore, ROS-induced autophagy contributes to fusion efficiency, but not the differentiation rate in mouse muscle stem cell-derived myoblasts [40]. Further studies on the mechanism underlying the regulation of myomaker expression by Nox4-dependent ROS including other molecules can provide direct and clear evidence for Nox4-mediated myoblast fusion through myomaker.

In conclusion, we showed that Nox4 expression enhances myoblast fusion without affecting differentiation during differentiation of primary myoblasts and C2C12 cells, and contributes to mouse skeletal muscle regeneration. One of the underlying mechanisms might be that Nox4-derived ROS contributes to the expression of myomaker, a fusogenic protein, leading to myoblast fusion. These findings provide a functional role of Nox4 in myoblast fusion during differentiation and skeletal muscle regeneration and will aid the development of therapeutic interventions in muscular diseases.

## Data Availability

The data used to support the findings of this study are available from the corresponding author upon request.

## Supplementary Materials

Supplementary Materials Supplementary Figure 1: Quantification of immunoblot signals is presented as the mean ± SD. (n = 3). Representative image is shown in Figure 2(f). Supplementary Figure 2: (a) Immunoblot analysis of primary myotubes cultured in DM for 48 h after cells were transfected with siCont or siNox4. (b) Quantification of immunoblot signals is presented. ^∗∗^*p* < 0.01 (*n* = 3). Supplementary Figure 3: Quantification of immunoblot signals is presented as the mean ± SD. (*n* = 3). Representative image is shown in Figures 3(b) and 3(f). (a), Nox4; (b), myosin heavy chain 3 (MHC); (c),myogenin. ^∗∗^*p* < 0.01, ^#^p < 0.001 (*n* = 3). Supplementary Figure 4: Nox4 rescue slightly promotes myoblast fusion. (a) Nox4-KO primary myoblasts infected with mock or Ad-Nox4 virus (MOI 10) for 24 h were incubated in differentiation medium (DM) for 3 days, and then stained with antibody against MHC (green) and DAPI for nucleus (blue). Scale bar, 50 *μ*m. For the experiment in a, differentiation index (b) was calculated as the percentage of nuclei in MHC-positive cells in total nuclei (*n* = 3). (c) The percentage of nuclei in MHC-positive cells with the indicated number of nuclei to total nuclei in MHC-positive cells were determined. ^∗^*p* < 0.05 (*n* = 3). ns, no significant difference. Supplementary Figure 5: Myomaker expression was suppressed in Nox4-KO mice. Mymk mRNA levels in TA muscles of WT and KO mice during regeneration after CTX injury (Figure 1(c)) was determined by RT-qPCR, using 36B4 for normalization. The following primers were used: Mymk 5′-ATCGCTACCAAGAGGCGTT-3′ and 5′-CACAGCACAGACAAACCAGG-3′; 36B4, 5′-AGATTCGGGATATGCTGTTGG-3′ and 5′-AAAGCCTGGAAGAAGGAGGTC-3′. ^∗∗^*p* < 0.01 (*n* = 3). Supplementary Figure 6: Knockdown of Nox4 has no effect on the mRNA expression of Minion, N-Wasp, and Rac1. After C2C12 cells were treated with either siCont or siNox4 for 24 h and incubated in DM for 3 days, the mRNA levels of Minion(a), N-Wasp (b), and Rac1 (c) during differentiation were analyzed by RT-qPCR, using 36B4 for normalizaion (*n* = 3). The following primers were used: Minion, 5′-GGACCACTCCCAGAGGAAGGA-3′ and 5′-GGACCGACGCCTGGACTAAC-3′; N-Wasp, 5′-AAGGATGGGAAACTATTGTGGGA-3′ and 5′-GACGGCCCAAAAGGTCTGTAA-3′; Rac1, 5′- CAATGCGTTCCCTGGAGAGTACA-3′ and 5′-ACGTCTGTTTGCGGGTAGGAGAG-3′; 36B4, 5′- AGATTCGGGATATGCTGTTGG-3′ and 5′-AAAGCCTGGAAGAAGGAGGTC-3′. Supplementary Figure 7: Quantification of immunoblot signals is presented as the mean ± SD.(*n* = 3). Representative image is shown in Figure 4(c). (a), Nox4; (b), myomaker. ^∗^*p* < 0.05, ^∗∗^*p* < 0.01, ^#^*p* < 0.001 (*n* = 3). Supplementary Figure 8: GKT137831 inhibits myoblast fusion in C2C12 cells. (a) C2C12 cells pretreated with DMSO or GKT137831 (GKT) for 24 h were cultured in DM for 3 days and then stained with antibody against MHC (green) and DAPI for nucleus (blue). Scale bar, 50 *μ*m. For the experiment in a, (b) the differentiation index was calculated as the percentage of nuclei in MHC-positive cells to total nuclei (*n* = 3). (c) The fusion index was determined as the percentage of nuclei in MHC-positive myotubes (≥10 nuclei) to total nuclei in MHC-positive myotubes. ^∗^*p* < 0.05 (*n* = 3). ns, no significant difference. Supplementary Figure 9: Quantification of immunoblot signals is presented as the mean ± SD. (*n* = 3). Representative image is shown in Figure 4(f). (a), Nox4; (b), myomaker. ^∗^*p* < 0.05, ^∗∗^*p* < 0.01 (*n* = 3). Supplementary Figure 10: Uncropped immunoblot image for myomaker in Figure 4(f) using primary antibody (Abcam 188300).

## References

[1] Loureiro A. C. C., do Rêgo-Monteiro I. C., Louzada R. A. (2016). Differential expression of NADPH oxidases depends on skeletal muscle fiber type in rats. *Oxidative Medicine and Cellular Longevity*.

[2] Regan J. N., Mikesell C., Reiken S. (2017). Osteolytic breast cancer causes skeletal muscle weakness in an immunocompetent syngeneic mouse model. *Frontiers in Endocrinology*.

[3] Sun Q. A., Hess D. T., Nogueira L. (2011). Oxygen-coupled redox regulation of the skeletal muscle ryanodine receptor-Ca^2+^ release channel by NADPH oxidase 4. *Proceedings of the National Academy of Sciences of the United States of America*.

[4] Liu X. H., Harlow L., Graham Z. A., Bauman W. A., Cardozo C. (2017). Spinal cord injury leads to hyperoxidation and nitrosylation of skeletal muscle ryanodine receptor-1 associated with upregulation of nicotinamide adenine dinucleotide phosphate oxidase 4. *Journal of Neurotrauma*.

[5] Handayaningsih A. E., Iguchi G., Fukuoka H. (2011). Reactive oxygen species play an essential role in IGF-I signaling and IGF-I-induced myocyte hypertrophy in C2C12 myocytes. *Endocrinology*.

[6] Cheng A. J., Bruton J. D., Lanner J. T., Westerblad H. (2015). Antioxidant treatments do not improve force recovery after fatiguing stimulation of mouse skeletal muscle fibres. *The Journal of Physiology*.

[7] Vogel J., Figueiredo de Rezende F., Rohrbach S., Zhang M., Schroder K. (2015). Nox4 is dispensable for exercise induced muscle fibre switch. *PLoS One*.

[8] Abmayr S. M., Pavlath G. K. (2012). Myoblast fusion: lessons from flies and mice. *Development*.

[9] Rochlin K., Yu S., Roy S., Baylies M. K. (2010). Myoblast fusion: when it takes more to make one. *Developmental Biology*.

[10] Gruenbaum-Cohen Y., Harel I., Umansky K. B. (2012). The actin regulator N-WASp is required for muscle-cell fusion in mice. *Proceedings of the National Academy of Sciences of the United States of America*.

[11] Vasyutina E., Martarelli B., Brakebusch C., Wende H., Birchmeier C. (2009). The small G-proteins Rac1 and Cdc42 are essential for myoblast fusion in the mouse. *Proceedings of the National Academy of Sciences of the United States of America*.

[12] Laurin M., Fradet N., Blangy A., Hall A., Vuori K., Côté J. F. (2008). The atypical Rac activator Dock180 (Dock1) regulates myoblast fusion in vivo. *Proceedings of the National Academy of Sciences of the United States of America*.

[13] Millay D. P., O'Rourke J. R., Sutherland L. B. (2013). Myomaker is a membrane activator of myoblast fusion and muscle formation. *Nature*.

[14] Millay D. P., Gamage D. G., Quinn M. E. (2016). Structure-function analysis of myomaker domains required for myoblast fusion. *Proceedings of the National Academy of Sciences of the United States of America*.

[15] Gamage D. G., Leikina E., Quinn M. E., Ratinov A., Chernomordik L. V., Millay D. P. (2017). Insights into the localization and function of myomaker during myoblast fusion. *Journal of Biological Chemistry*.

[16] Shi J., Cai M., Si Y., Zhang J., Du S. (2018). Knockout of myomaker results in defective myoblast fusion, reduced muscle growth and increased adipocyte infiltration in zebrafish skeletal muscle. *Human Molecular Genetics*.

[17] Luo W., Li E., Nie Q., Zhang X. (2015). Myomaker, regulated by MYOD, MYOG and miR-140-3p, promotes chicken myoblast fusion. *International Journal of Molecular Sciences*.

[18] Di Gioia S. A., Connors S., Matsunami N. (2017). A defect in myoblast fusion underlies Carey-Fineman-Ziter syndrome. *Nature Communications*.

[19] Leikina E., Gamage D. G., Prasad V. (2018). Myomaker and myomerger work independently to control distinct steps of membrane remodeling during myoblast fusion. *Developmental Cell*.

[20] Zhang Q., Vashisht A. A., O’Rourke J. (2017). The microprotein minion controls cell fusion and muscle formation. *Nature Communications*.

[21] Quinn M. E., Goh Q., Kurosaka M. (2017). Myomerger induces fusion of non-fusogenic cells and is required for skeletal muscle development. *Nature Communications*.

[22] Bi P., Ramirez-Martinez A., Li H. (2017). Control of muscle formation by the fusogenic micropeptide myomixer. *Science*.

[23] He J., Wang F., Zhang P. (2017). miR-491 inhibits skeletal muscle differentiation through targeting myomaker. *Archives of Biochemistry and Biophysics*.

[24] Lee J. H., Joo J. H., Kim J. (2013). Interaction of NADPH oxidase 1 with toll-like receptor 2 induces migration of smooth muscle cells. *Cardiovascular Research*.

[25] Lee K. P., Shin Y. J., Panda A. C. (2015). miR-431 promotes differentiation and regeneration of old skeletal muscle by targeting Smad4. *Genes and Development*.

[26] Hindi S. M., Shin J., Gallot Y. S. (2017). MyD88 promotes myoblast fusion in a cell-autonomous manner. *Nature Communications*.

[27] Acharya S., Peters A. M., Norton A. S., Murdoch G. K., Hill R. A. (2013). Change in Nox4 expression is accompanied by changes in myogenic marker expression in differentiating C2C12 myoblasts. *Pflügers Archiv*.

[28] Ganassi M., Badodi S., Ortuste Quiroga H. P., Zammit P. S., Hinits Y., Hughes S. M. (2018). Myogenin promotes myocyte fusion to balance fibre number and size. *Nature Communications*.

[29] Zhang W., Roy S. (2017). Myomaker is required for the fusion of fast-twitch myocytes in the zebrafish embryo. *Developmental Biology*.

[30] Zhong X., Wang Q. Q., Li J. W., Zhang Y. M., An X. R., Hou J. (2017). Ten-Eleven Translocation-2 (Tet2) Is Involved in Myogenic Differentiation of Skeletal Myoblast Cells _in Vitro_. *Scientific Reports*.

[31] Shen X., Collier J. M., Hlaing M. (2003). Genome‐wide examination of myoblast cell cycle withdrawal during differentiation. *Developmental Dynamics*.

[32] Ito N., Ruegg U. T., Kudo A., Miyagoe-Suzuki Y., Takeda S. (2013). Activation of calcium signaling through Trpv1 by nNOS and peroxynitrite as a key trigger of skeletal muscle hypertrophy. *Nature Medicine*.

[33] Matsushima S., Kuroda J., Ago T. (2013). Increased oxidative stress in the nucleus caused by Nox4 mediates oxidation of HDAC4 and cardiac hypertrophy. *Circulation Research*.

[34] Somanna N. K., Valente A. J., Krenz M., Fay W. P., Delafontaine P., Chandrasekar B. (2016). The Nox1/4 Dual Inhibitor GKT137831 or Nox4 Knockdown Inhibits Angiotensin‐II‐Induced Adult Mouse Cardiac Fibroblast Proliferation and Migration. AT1 Physically Associates With Nox4. *Journal of Cellular Physiology*.

[35] Zakharova V. V., Dib C., Saada Y. B. (2016). Uncoupling of oxidative phosphorylation and antioxidants affect fusion of primary human myoblasts in vitro. *Biopolymers and Cell*.

[36] Xu M., Li X. X., Chen Y., Pitzer A. L., Zhang Y., Li P. L. (2014). Enhancement of dynein‐mediated autophagosome trafficking and autophagy maturation by ROS in mouse coronary arterial myocytes. *Journal of Cellular and Molecular Medicine*.

[37] He C., Zhu H., Zhang W. (2013). 7-Ketocholesterol induces autophagy in vascular smooth muscle cells through Nox4 and Atg4B. *American Journal of Pathology*.

[38] Sciarretta S., Zhai P., Shao D. (2013). Activation of NADPH oxidase 4 in the endoplasmic reticulum promotes cardiomyocyte autophagy and survival during energy stress through the protein kinase RNA-activated-like endoplasmic reticulum kinase/eukaryotic initiation factor 2*α*/activating transcription factor 4 pathway. *Circulation Research*.

[39] Luo X., Yang D., Wu W. (2018). Critical role of histone demethylase Jumonji domain-containing protein 3 in the regulation of neointima formation following vascular injury. *Cardiovascular Research*.

[40] Fortini P., Ferretti C., Iorio E. (2016). The fine tuning of metabolism, autophagy and differentiation during _in vitro_ myogenesis. *Cell Death & Disease*.

