# Supplementary material for: NADPH Oxidase 4 Contributes to Myoblast Fusion and Skeletal Muscle Regeneration
Source: Oxid Med Cell Longev. 2019 Nov 18;2019:3585390. doi: 10.1155/2019/3585390 (PMC6885834; doi:10.1155/2019/3585390)
Supplement: Supplementary Materials — Supplementary Figure 1: Quantification of immunoblot signals is presented as the mean ± SD. (n = 3). Representative image is shown in Figure 2(f). Supplementary Figure 2: (a) Immunoblot analysis of primary myotubes cultured in DM for 48 h after cells were transfected with siCont or siNox4. (b) Quantification of immunoblot signals is presented. ∗∗p < 0.01 (n = 3). Supplementary Figure 3: Quantification of immunoblot signals is presented as the mean ± SD. (n = 3). Representative image is shown in Figures 3(b) and 3(f). (a), Nox4; (b), myosin heavy chain 3 (MHC); (c),myogenin. ∗∗p < 0.01, #p < 0.001 (n = 3). Supplementary Figure 4: Nox4 rescue slightly promotes myoblast fusion. (a) Nox4-KO primary myoblasts infected with mock or Ad-Nox4 virus (MOI 10) for 24 h were incubated in differentiation medium (DM) for 3 days, and then stained with antibody against MHC (green) and DAPI for nucleus (blue). Scale bar, 50 μm. For the experiment in a, differentiation index (b) was calculated as the percentage of nuclei in MHC-positive cells in total nuclei (n = 3). (c) The percentage of nuclei in MHC-positive cells with the indicated number of nuclei to total nuclei in MHC-positive cells were determined. ∗p < 0.05 (n = 3). ns, no significant difference. Supplementary Figure 5: Myomaker expression was suppressed in Nox4-KO mice. Mymk mRNA levels in TA muscles of WT and KO mice during regeneration after CTX injury (Figure 1(c)) was determined by RT-qPCR, using 36B4 for normalization. The following primers were used: Mymk 5′-ATCGCTACCAAGAGGCGTT-3′ and 5′-CACAGCACAGACAAACCAGG-3′; 36B4, 5′-AGATTCGGGATATGCTGTTGG-3′ and 5′-AAAGCCTGGAAGAAGGAGGTC-3′. ∗∗p < 0.01 (n = 3). Supplementary Figure 6: Knockdown of Nox4 has no effect on the mRNA expression of Minion, N-Wasp, and Rac1. After C2C12 cells were treated with either siCont or siNox4 for 24 h and incubated in DM for 3 days, the mRNA levels of Minion(a), N-Wasp (b), and Rac1 (c) during differentiation were analyzed by RT-qPCR, using 36B4 for [file 3585390.f1.pdf]

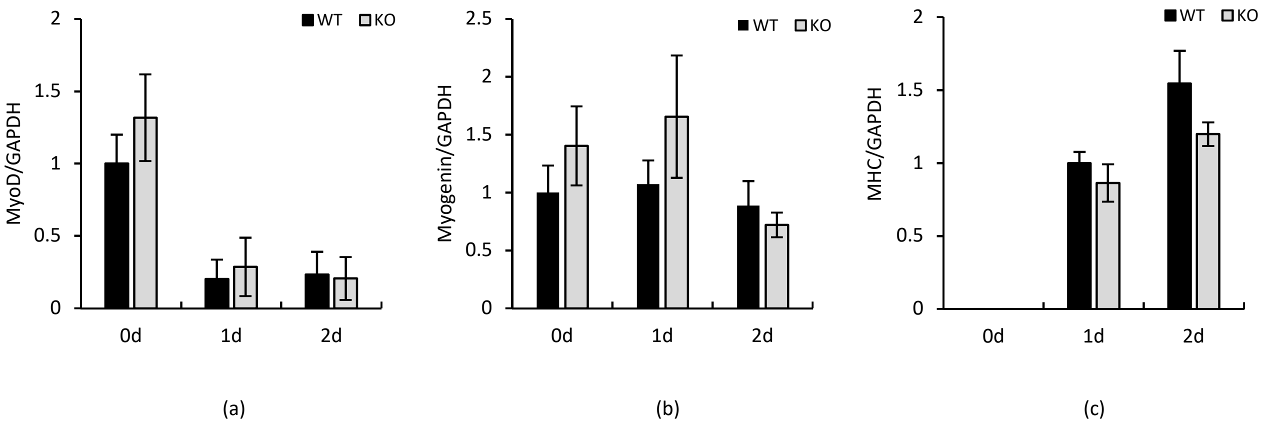

Supplementary figure 1: Quantification of immunoblot signals is presented as the mean ± SD. (n = 3). Representative image is shown in Figure 2(f).

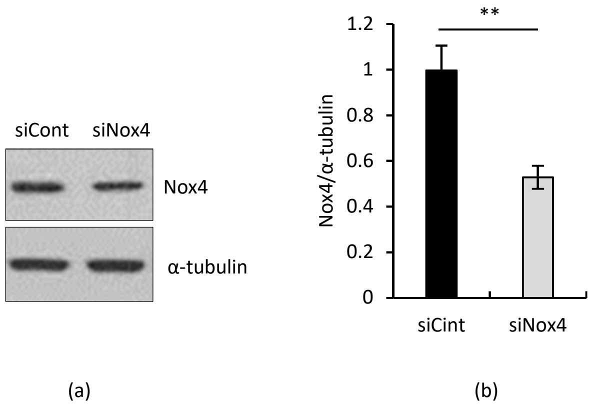

Supplementary figure 2: (a) Immunoblot analysis of primary myotubes cultured in DM for 48 h after cells were transfected with siCont or siNox4. (b) Quantification of immunoblot signals is presented. $^{**}p < 0.01$ ($n = 3$).

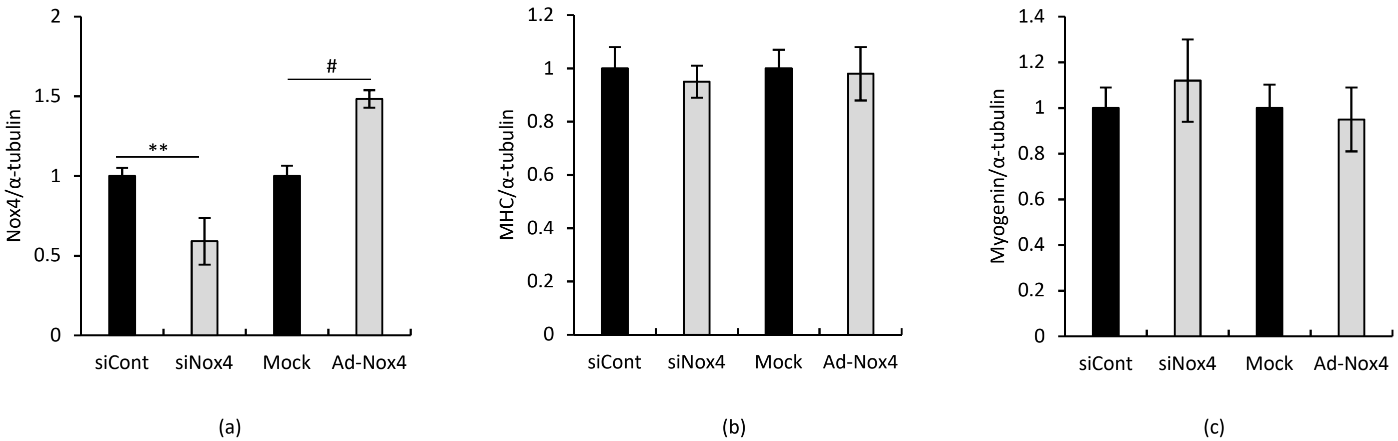

Supplementary figure 3: Quantification of immunoblot signals is presented as the mean ± SD. (n = 3). Representative image is shown in Figures 3(b) and 3(f). (a), Nox4; (b), myosin heavy chain 3 (MHC); (c), myogenin. $^{**}p < 0.01$, $^{\#}p < 0.001$ (n = 3).

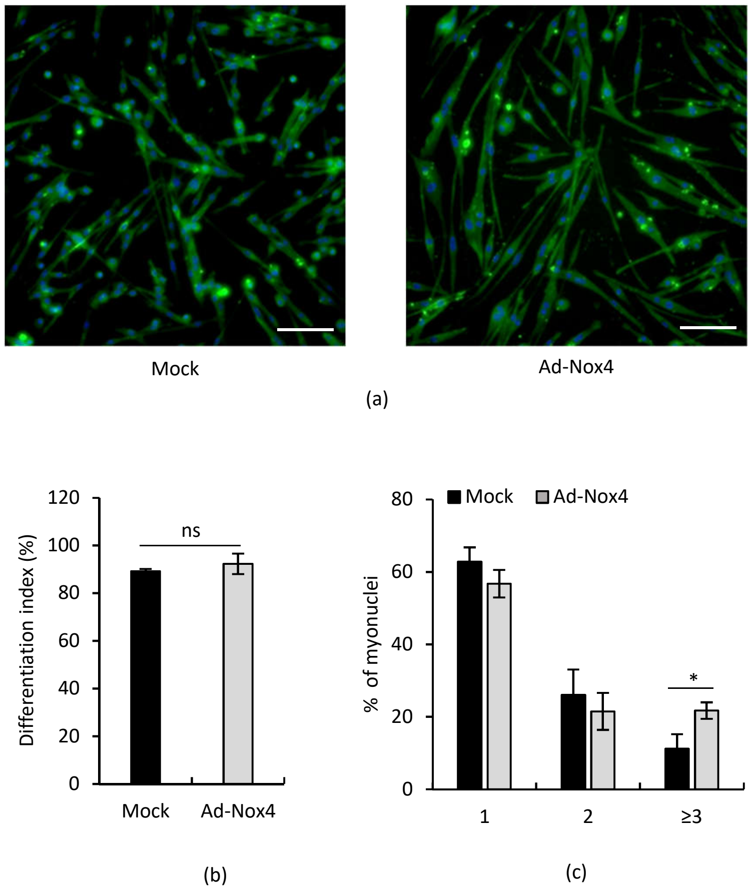

Supplementary figure 4: Nox4 rescue slightly promotes myoblast fusion. (a) *Nox4*-KO primary myoblasts infected with mock or Ad-Nox4 virus (MOI 10) for 24 h were incubated in differentiation medium (DM) for 3 days, and then stained with antibody against MHC (green) and DAPI for nucleus (blue). Scale bar, 50 µm. For the experiment in a, differentiation index (b) was calculated as the percentage of nuclei in MHC-positive cells in total nuclei (n = 3). (c) The percentage of nuclei in MHC-positive cells with the indicated number of nuclei to total nuclei in MHC-positive cells were determined. $^{*}p < 0.05$ (n = 3). ns, no significant difference.

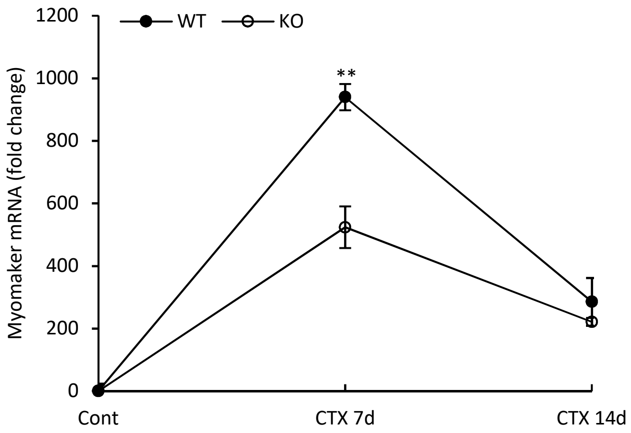

Supplementary figure 5: Myomaker expression was suppressed in Nox4-KO mice. *Mymk* mRNA levels in TA muscles of WT and KO mice during regeneration after CTX injury (Figure 1(c)) was determined by RT-qPCR, using *36B4* for normalization. The following primers were used: *Mymk* 5′-ATCGCTACCAAGAGGCGTT-3′ and 5′-CACAGCACAGACAAACCAGG-3′; *36B4*, 5′-AGATTCGGGATATGCTGTTGG-3′ and 5′-AAAGCCTGGAAGAAGGAGGTC-3′. $^{**}p < 0.01$ ($n = 3$).

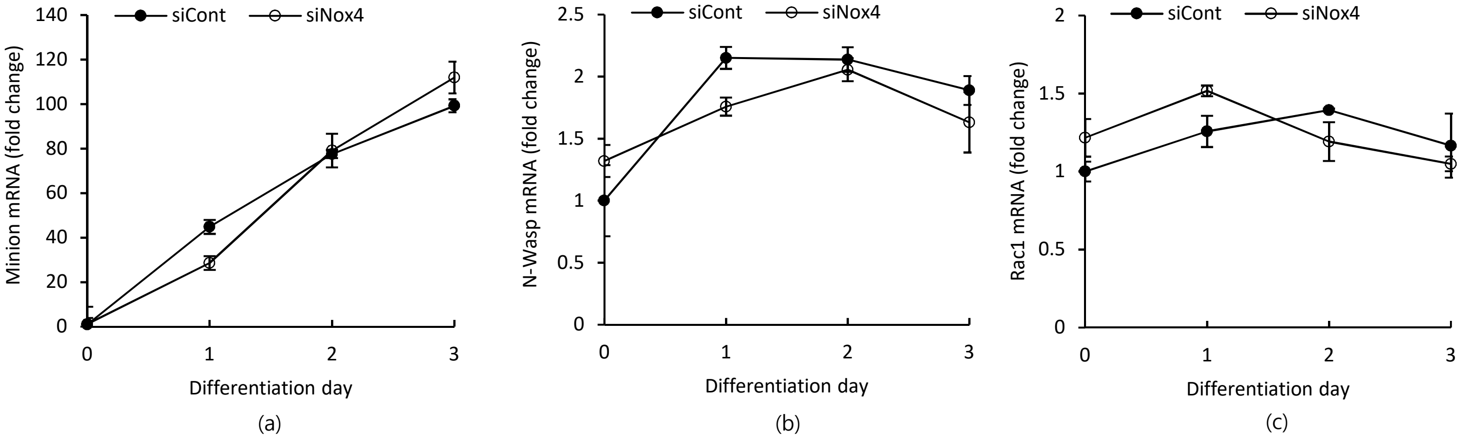

Supplementary figure 6: Knockdown of *Nox4* has no effect on the mRNA expression of *Minion*, *N-Wasp*, and *Rac1*. After C2C12 cells were treated with either siCont or siNox4 for 24 h and incubated in DM for 3 days, the mRNA levels of *Minion* (a), *N-Wasp* (b), and *Rac1* (c) during differentiation were analyzed by RT-qPCR, using *36B4* for normalizaion (n = 3). The following primers were used: *Minion*, 5′-GGACCACTCCCAGAGGAAGGA-3′ and 5′-GGACCGACGCCTGGACTAAC-3′; *N-Wasp*, 5′-AAGGATGGGAAACTATTGTGGGA-3′ and 5′-GACGGCCCAAAAGGTCTGTAA-3′; *Rac1*, 5′-CAATGCGTTCCCTGGAGAGTACA-3′ and 5′-ACGTCTGTTTGCGGGTAGGAGAG-3′; *36B4*, 5′-AGATTCGGGATATGCTGTTGG-3′ and 5′-AAAGCCTGGAAGAAGGAGGTC-3′.

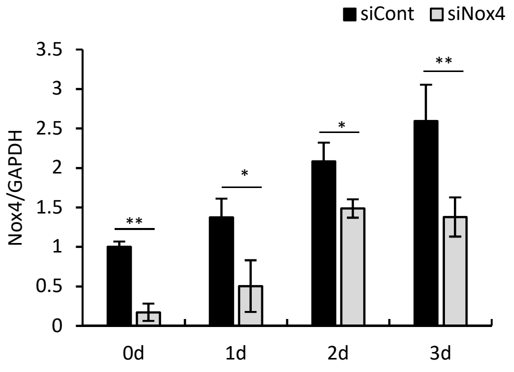

(a)

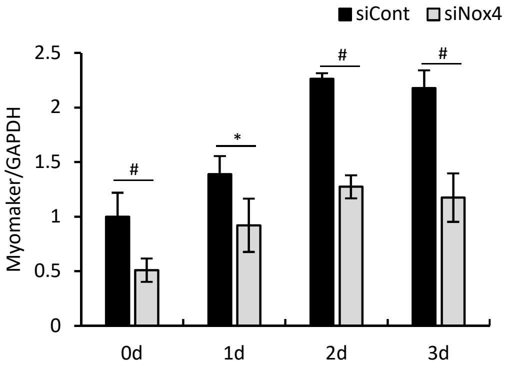

(b)

Supplementary figure 7: Quantification of immunoblot signals is presented as the mean ± SD. (n = 3). Representative image is shown in Figure 4(c). (a), Nox4; (b), myomaker. $^{*}p < 0.05$, $^{**}p < 0.01$, $^{\#}p < 0.001$ (n = 3).

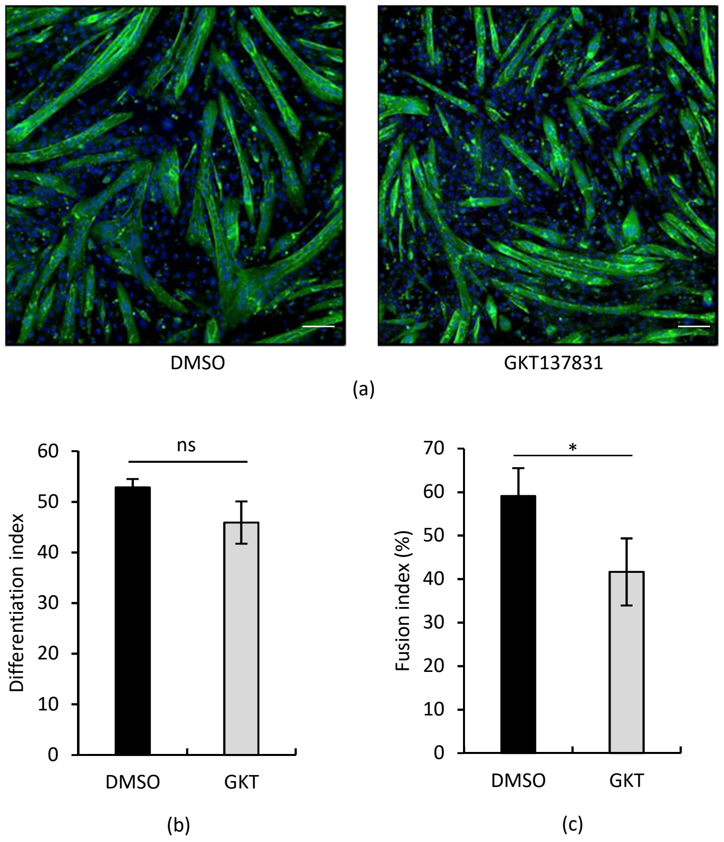

Supplementary figure 8: GKT137831 inhibits myoblast fusion in C2C12 cells. (a) C2C12 cells pretreated with DMSO or GKT137831 (GKT) for 24 h were cultured in DM for 3 days and then stained with antibody against MHC (green) and DAPI for nucleus (blue). Scale bar, 50 µm. For the experiment in a, (b) the differentiation index was calculated as the percentage of nuclei in MHC-positive cells to total nuclei (*n* = 3). (c) The fusion index was determined as the percentage of nuclei in MHC-positive myotubes (≥10 nuclei) to total nuclei in MHC-positive myotubes. $^{*}p < 0.05$ ($n = 3$). ns, no significant difference.

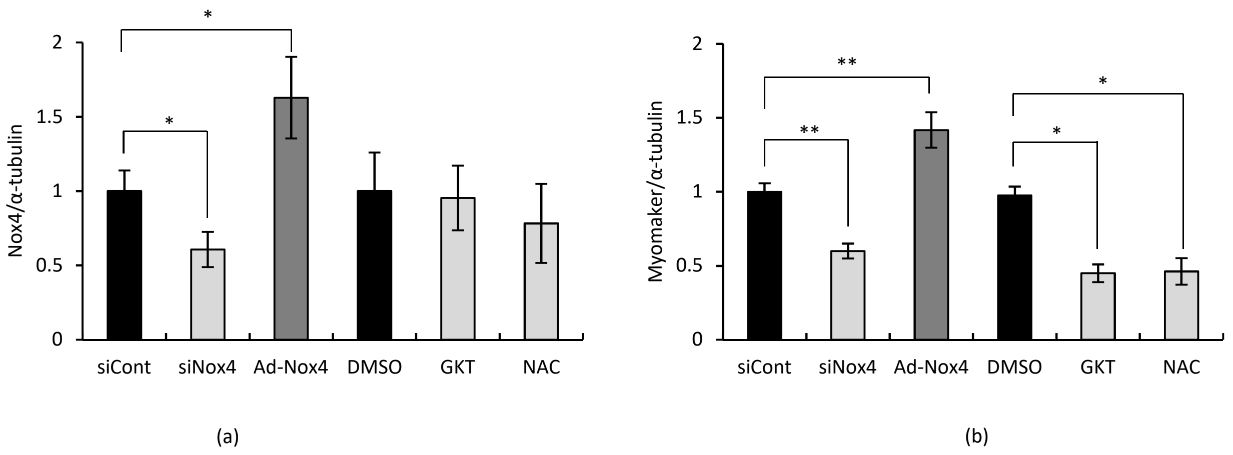

Supplementary figure 9: Quantification of immunoblot signals is presented as the mean ± SD. (n = 3). Representative image is shown in Figure 4(f). (a), Nox4; (b), myomaker. $^{*}p < 0.05$, $^{**}p < 0.01$ (n = 3).

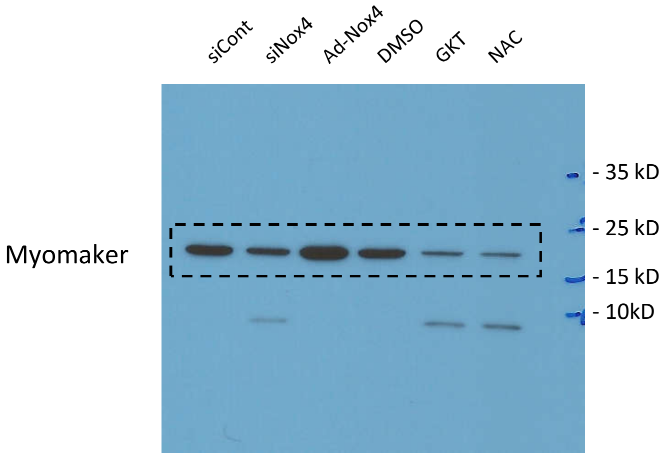

Supplementary figure 10: Uncropped immunoblot image for myomaker in Figure 4(f) using primary antibody (Abcam 188300).
